# Supplementary material for: Encapsulation and Characterization of Nanoemulsions Based on an Anti-oxidative Polymeric Amphiphile for Topical Apigenin Delivery
Source: Polymers (Basel). 2021 Mar 25;13(7):1016. doi: 10.3390/polym13071016 (PMC8037426; doi:10.3390/polym13071016)
Supplement: Supplementary file 1 [file polymers-13-01016-s001.pdf]

Supporting Information

Encapsulation and characterization of nanoemulsions based on an anti-oxidative polymeric amphiphile for topical apigenin delivery

Tzung-Han Chou[1,*], Daniel Setiyo Nugroho[1], Jia-Yaw Chang[2], Yu-Shen Cheng[1], Chia-Hua Liang[3] and Ming-Jay Deng[4]

[1] Department of Chemical and Materials Engineering, National Yunlin University of Science and Technol-ogy, Yunlin 64022, Taiwan; chouth@yuntech.edu.tw

[2] Department of Chemical Engineering, National Taiwan University of Science and Technology, Taipei 10607, Taiwan; jychang@mail.ntust.edu.tw

[3] Department of Cosmetic Science, Chia Nan University of Pharmacy and Science, Tainan 717, Taiwan; tinna_ling@mail.cnu.edu.tw

[4] Department of Applied Chemistry, Providence University, 200 Taiwan Boulevard, Sec. 7, Taichung, 43301, Taiwan; dengmj1020@pu.edu.tw

*Correspondence: Author: Tel. (+) 886-5-5342601 ext. 4625, Fax. (+) 886-5-5312071. E-mail: chouth@yuntech.edu.tw

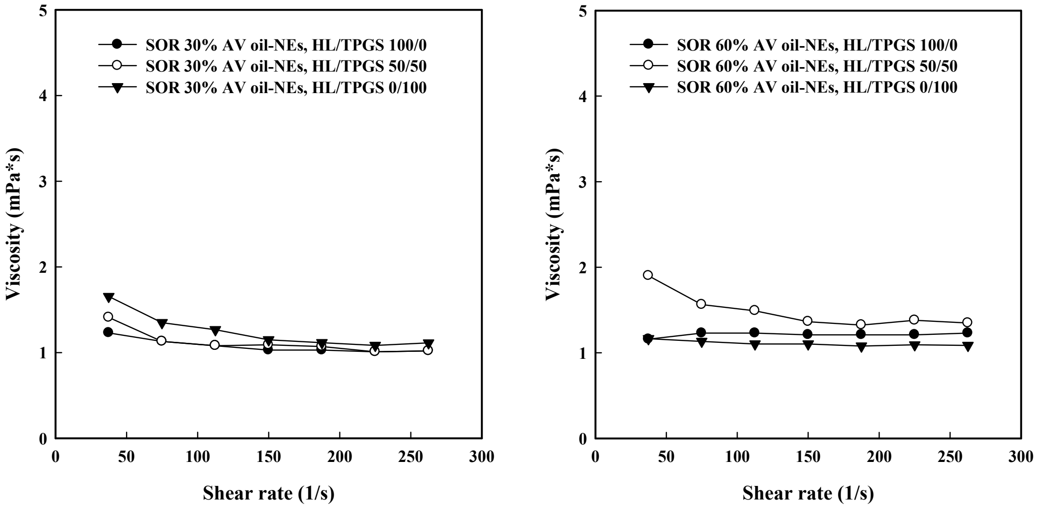

Figure S1. The viscosity of fresh AV oil-NEs with SOR 30 and 60%, oil content 1%, and different surfactant ratios of HL to TPGS as a function of the shear rate at 25°C.

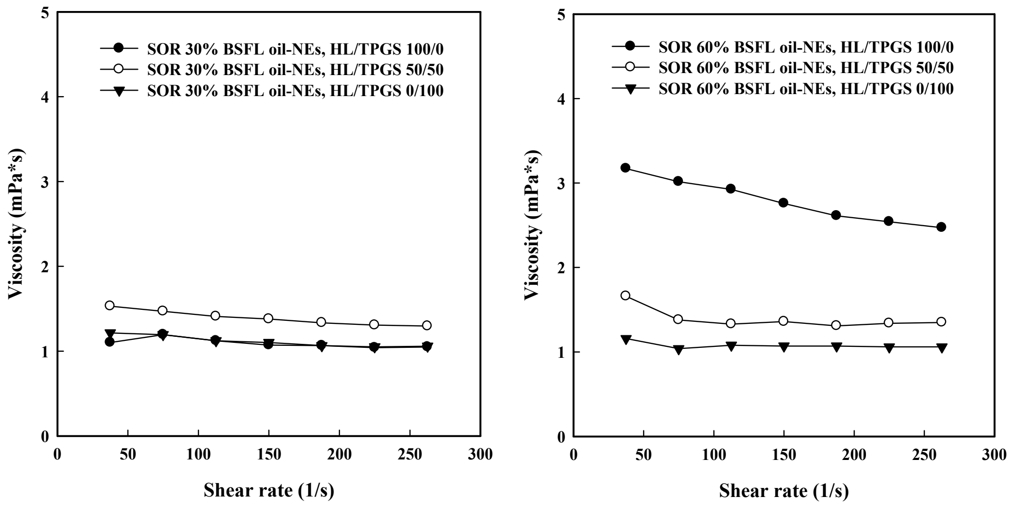

Figure. S2. The viscosity of fresh BSFL oil-NEs with SOR 30 and 60%, oil content 1%, and different surfactant ratios of HL to TPGS as a function of the shear rate at 25°C.
